# *Aedes aegypti* microRNA, miR-2944b-5p interacts with 3'UTR of chikungunya virus and cellular target vps-13 to regulate viral replication

**DOI:** 10.1371/journal.pntd.0007429

**Published:** 2019-06-05

**Authors:** Sunil Kumar Dubey, Jatin Shrinet, Sujatha Sunil

**Affiliations:** Vector Borne Diseases Group, International Centre for Genetic Engineering and Biotechnology (ICGEB), Aruna Asaf Ali Marg, New Delhi, India; Fundacao Oswaldo Cruz, BRAZIL

## Abstract

**Background:**

RNA interference is among the most important mechanisms that serve to restrict virus replication within mosquitoes, where microRNAs (miRNAs) are important in regulating viral replication and cellular functions. These miRNAs function by binding to complementary sequences mostly in the untranslated regions of the target. Chikungunya virus (CHIKV) genome consists of two open reading frames flanked by 5′ and 3′ untranslated regions on the two sides. A recent study from our laboratory has shown that *Aedes* miRNAs are regulated during CHIKV infection. The present study was undertaken to further understand the role of these miRNAs in CHIKV replication.

**Methods/Findings:**

We observe that miR-2944b-5p binds to the 3′ untranslated region of CHIKV and the binding is abated when the binding sites are abolished. Loss-of-function studies of miR-2944b-5p using antagomirs, both *in vitro* and *in vivo*, reveal an increase in CHIKV viral replication, thereby directly implying a role of miR-2944b-5p in CHIKV replication. We further showed that the mitochondrial membrane potential of the mosquito cells is maintained by this miRNA during CHIKV replication, and cellular factor vps-13 plays a contributing role.

**Conclusions:**

Our study has opened new avenues to understand vector-virus interactions and provides novel insights into CHIKV replication in *Aedes aegypti*. Furthermore, our study has shown miR-2944b-5p to be playing role, where one of its target vps-13 also contributes, in maintaining mitochondrial membrane potential in *Aedes aegypti*.

## Author summary

*Aedes aegypti* mosquito transmits pathogenic viruses like chikungunya virus (CHIKV). Inside the vector, the virus replicates in a way so that it is able to survive within the mosquito without causing damage to it. However, once in the mammalian host, it becomes pathogenic and induces death to the infected cells. Amongst several mosquito specific factors that allows or rejects the virus survival, microRNAs play a decisive role. In several studies, miRNAs have shown to be playing role in controlling virus replication either by binding to viral genome or to suppress the expression of any host factor. In the present study, we identified an *Aedes* miRNA, miR-2944b-5p, which binds to 3'UTR of CHIKV and regulates the replication of the virus in the mosquito. Analysis of the mode of action of this regulation revealed that miR-2944b-5p played a role in maintaining mitochondrial membrane potential during CHIKV replication by targeting cellular factor vps-13.

## Introduction

Mosquitoes aid the transmission of several pathogenic viruses collectively called arboviruses that mainly belong to the Flaviviridae and Togaviridae families. These are single-stranded RNA viruses that replicate actively within the mosquitoes and reach titers that are then transmitted by the vector to a vertebrate host upon a blood meal. However, mosquitoes employ innate immune responses against arboviruses, thereby restricting their replication in the vector [1].

Among the innate immune responses exhibited by mosquitoes, RNA interference (RNAi) is one of the important mechanisms that play a role in restricting virus replication in mosquitoes [2–6]. This phenomenon functions through different pathways aided by a variety of small-RNA population—small interfering RNAs, virus-derived interfering RNAs, and microRNAs (miRNAs)—that bind to different RNA-binding proteins and are processed in the RNA interference silencing complex (RISC), resulting in the degradation/repression of target molecules [7]. Studies have shown that there is a distinct crosstalk amongst the RNA-silencing pathways aimed to provide an effective RNA-silencing response [8].

miRNAs are short, generally 21–24 bp long single-stranded RNAs, that participate in the degradation of mRNA, thereby inhibiting its translation. The degradation is initiated by the annealing of miRNAs directly to seed sequences in the 3′ untranslated region (UTR) of the mRNA and by the recruitment of specific host proteins [9]. Several studies have reported that cellular miRNAs of the host inhibit the replication of several viruses such as human immunodeficiency virus (HIV), enteroviruses, and influenza virus by binding to coding region of viral genome either directly or indirectly and inhibiting its translation [10–14]. Similarly, several viruses have shown to utilize host miRNAs to their advantage, either to escape host surveillance and maintain viral latency or to promote viral replication [15,16].

Chikungunya virus (CHIKV) is a positive sense single-stranded RNA virus of genus *Alphavirus*, belonging to family Togaviridae. Transmitted by *Aedes aegypti* and *Aedes albopictus* mosquitoes, this virus infects humans, causing acute febrile illness and severe arthralgia. The CHIKV genome contains two open reading frames encoding nonstructural and structural polyproteins and is flanked by a 76 nt long 5′ UTR and a 3′ UTR that ranges between 450–900 nt depending on the lineage, on either side of the open reading frames and an internal subgenomic 5’ UTR, 48 nt long [17]. Recent studies have identified sequence elements involved in viral replication and host interactions, thereby emphasizing the importance of the UTRs of the CHIKV genome in its replication and fitness, both in the vector and in the host [18].

One of the most important interactions between the host/vector and viruses is the interaction of cellular miRNAs with viral UTRs. Among the other interactions that can potentially affect virus replication, are interactions involving the cellular targets of miRNAs that can be beneficially hijacked by the viruses for their own advantages. Whereas there are reports of host miRNAs binding to viral RNAs and restricting viral growth [19], several studies show that host miRNAs may bind to the viral genome to promote viral replication. In one such study HCV miR-122 has been shown to play this role [15,20]. Likewise, many studies have shown that miRNAs targets aid in increase or decrease viral replication thereby posing as host factors [21,22]. Many studies identified a repertoire of mosquito miRNAs that are regulated upon viral infection; amongst which, miR-2944b-5p and miR-2b have shown to have an impact on the pathogenesis of several mosquito-borne pathogens [23,24].

We undertook the present study to further understand the role of these two miRNAs in CHIKV replication in *Ae*. *aegypti* and cellular targets affecting CHIKV replication. We performed the luciferase assay and observed that miR-2944b-5p and miR-2b bind to the 3′ UTR of CHIKV and that this binding is abated when the binding sites are abolished. Loss-of-function studies of miR-2944b-5p and miR-2b using antagomirs, both *in vitro*, reveal an increase in CHIKV replication prominently in the case of miR-2944b-5p, thereby directly implying a role of miR-2944b-5p in CHIKV replication. Analysis of *Ae*. *aegypti* cellular targets for miR-2944b-5p reveals that vacuolar protein sorting (vps-13) is a target and plays a role in regulating CHIKV replication in *Ae*. *aegypti* cells. Our findings put together provide evidence that CHIKV may be using miR-2944b-5p and its target vps-13 to maintain cellular mitochondrial membrane potential (MMP)/integrity during its replication in mosquito cells. We propose that this could be an approach of the virus to survive in the mosquito cells.

## Materials and methods

### Ethics statement

Scientific and ethical approval to carry out this study was obtained from the ICGEB-Institutional Animal Ethics Committee (ICGEB-IAEC). The IAEC approval number is ICGEB/IAEC/280718/VBD-4. The ICGEB-IAEC adheres to national guidelines by Committee for the purpose of control and supervision of experiments on animals (CPCSEA).

### Cell lines

The Aag2 cell line (A kind gift from Prof. Alain Kohl, University of Glasgow) was cultured in Schneider Insect Media (HiMedia) at 28˚C. The HEK-293T (American Type Culture Collection-ATCC) cell lines were cultured in DMEM (HiMedia) at 37˚C. All cell lines were supplemented with 10% fetal bovine serum (FBS) (HiMedia) and antibiotics (10,000 U/ml penicillin and 10,000 μg/ml streptomycin; CellClone). CHIKV strain used for infection studies was from a clinical isolate IND-10-DEL1 (ECSA) obtained during a recent outbreak [25].

### Virus titrations

CHIKV virus was propagated in Vero cells and virus contatining supernatant was collected after 72 hrs and aliquoted to store at -80°C. The infectious virus titer in supernatant was determined by standard plaque assay on Vero cells [26].

### miRNA and transcript expression profiling using qRT-PCR

For miRNA expression profiling, RNA was isolated using Trizol method. The concentration of RNA was determined using a NanoDrop 2000 spectrophotometer (Thermo Scientific). The purity of RNA was assessed by calculating the ratio absorbance at 260 and 280 nm. All RNA preparations had a ratio of absorbance (260/280 nm) greater than 1.8. cDNA synthesis and PCR assay were performed using a commercial kit (NCode miRNA first-strand cDNA synthesis and qRT-PCR kit; Invitrogen) per the manufacturer’s instructions. The initial miRNA concentration was set at 500 ng. An aliquot (2.5 μl) of the cDNA was used for qRT-PCR using specific forward primers (identical to entire miRNA sequence) for the selected miRNA and the reverse primer was a universal qPCR primer. All the reactions for miRNAs and transcripts analysis were performed in triplicate by qRT-PCR with a PikoReal 96 Real-Time PCR system (Thermo Scientific). RNA for transcript expressions was used from the initial RNA isolated for miRNA using the trizol following manufacturer instructions. Expression levels of the selected miRNAs and transcripts (viral genomic RNA, vps-13 mRNA) were compared with those in controls after normalization against the housekeeping gene (5.8s) **(S1 Table)** using the cycle threshold (ΔΔ*CT*) method.

### miRNAs and 3′ UTR cloning

All mature miRNAs were mapped against the *Ae*. *aegypti* genome using Bowtie, and 250 flanking regions from each side were extracted using in-house Perl scripts. Pre-miRNAs were PCR-amplified from the *Ae*. *aegypti* mosquito and cloned in a pmR-mCherry vector (Clontech Lab). The 3′ UTR of CHIKV and putative cellular targets (AAEL011195, AAEL008432, and vps-13) were cloned in a pmir-GLO vector (Promega).

### Site-directed mutagenesis

The miR-2944b-5p and miR-2b binding sites at the 3′ UTR of CHIKV were amplified with primers containing desired mutations at seed region for the binding sites of miR-2944b-5p and miR-2b and cloned into the pmirGLO vector for the luciferase assay.

### Luciferase reporter assay

Plasmids expressing UTRs of CHIKV and cellular factors (AAEL011195, AAEL008432, and vps-13) and miRNAs were transfected in HEK-293T cells using JetPRIME Transfection reagent as per the manufacturer’s instructions. Luciferase activities were measured using a luminometer according to the manufacturer’s instructions (Glomax20/20 luminometer, Promega) 24 h post-transfection using the Dual-Glo luciferase reporter assay system (Promega). Renilla luciferase activity was normalized using firefly luciferase activity for each sample. Empty pmirGLO vector along with miR-2944b-5p cloned in pmR-mCherry vector was taken as control for miRNA miR-2944b-5p binding to the UTR's of cellular factors (AAEL011195, AAEL008432, and vps-13). Empty pmR-mCherry vector along with CHIKV 3'UTR pmirGLO vector was used as a control in case of miRNA miR-2944b-5p and miR-2b binding to the CHIKV 3'UTR.

### Double stranded RNA (dsRNA) preparation

For dsRNA preparation, the vps-13 was cloned in pGEMT-easy vector (Promega, USA) and was *in vitro* transcribed with T7 and SP6 polymerase using Riboprobe combination kit (Promega, USA). As a mock or control, dsRNA for gfp was used. The dsRNAs were purified using Trizol (Invitrogen, USA) method described earlier and stored till further use.

### Antimir(Antagomir) and dsRNA transfected virus infection in Aag2 cell line

Antagomirs (miRNA inhibitors) for miRNAs were purchased from Ambion (miRNA Inhibitors are chemically modified, single stranded nucleic acids designed to specifically bind to and inhibit endogenous microRNA molecules). The Aag2 cells were grown in 6-well plates and allowed to reach a confluence of 70%. At this point, the cells were transfected with 100 picomoles of the antigomirs (Antimir-2b, Antimir-2944b-5p, and a scrambled miRNA, that served as negative control) using Cellfectin II 2000 (Invitrogen). In case of the dsRNA, 2μg of RNA was transfected in each well as per manufacturer's protocol. The gfp dsRNA was also transfected that served as the negative control. After 24 hrs, the cells were infected with media containing virus with MOI 1.

### miR-2944b-5p target prediction

UTR sequences of *Ae*. *aegypti* genes were downloaded from VectorBase and targets of miR-2944b-5p were predicted using RNAhybrid tool. The targets were filtered on the basis of complementarities of the miRNAs with the targets and energy of the miRNA:target i.e, ≤ -30 Kcal/mol.

### Antagomir and dsRNA nano-injection in mosquitoes

Female mosquitoes (4–5 days old) were divided into three batches of 20 mosquitoes each. The first batch was injected with 69 nl of PBS. The second and third batches of mosquitoes were injected with 69 nl of 100 μM negative control RNA (scrambled miRNA) and miR-2944b-5p antagomir respectively. For dsRNA study, 800ng of dsRNA was injected in each mosquito. The mosquitoes were allowed to recover for 24 hrs and were fed on an infected meal. The whole mosquitoes were then stored 24 hrs post-feeding in Trizol at −80°C until RNA extraction. Knockdown of miRNA expression post-injection was checked by miRNA qRT-PCR and for CHIKV genomic RNA, primers for E1 gene were used.

### Mosquito feeding with infectious meal

Four-day-old female *Ae*. *aegypti* mosquitoes were orally fed with blood containing CHIKV virus using a glass blood feeder. The infectious blood meal contained 50% defibrinated blood (Source: Balb/C Mice), 10% FBS, and 40% DMEM containing freshly propagated purified virus to result in an estimated titer of 10^6^ plaque-forming units per milliliter.

### Measurement of *Aedes* mitochondrial membrane potential (MMP)

The 20×10^4^ cells were seeded on autoclaved coverslips one per well in a 24-well and were transfected with miR-2944b-5p inhibitor, vps-13 dsRNA, followed by CHIKV infection (MOI 1). CHIKV infection was done 24 hrs post transfection and after 24 hrs post infection cells were subsequently stained with JC-1(Dye to measure mitochondrial potential) (BD MitoScreen JC-1 Kit, Becton Dickinson, USA) for 15 min at 37°C and nuclei were stained with DAPI. The MMP was detected using microscopy experiments. The ratio JC-1 red fluorescence and JC-1 green fluorescence was used to measure the MMP or total polarized mitochondria in cells. NIS-Elements (Nikon) was used for acquiring the images as Z-stacks using a Nikon EclipseTi-E laser scanning confocal microscope equipped with a 60×/1.4 NA Plan Apochromat DIC objective.

### Statistical analysis

Data were shown as standard error of the mean. The experiments were repeated at least thrice and each experiment included at least three treatments. The error bars generated were from experimental replicates. Statistical analysis of experimental data was performed using GraphPad Prism (version5) using Student's *t*-test when comparing two conditions. Data from different treatments were subjected to an analysis of variance (ANOVA). For multiple comparisons, Tukey’s test or Dunnett’s test was performed. Values of *P*<0.05 represented with an asterisk were considered significant.

## Results

### *Ae*. *aegypti* miRNAs miR-2944b-5p and miR-2b bind to CHIKV 3′ UTR

**Fig 1:**
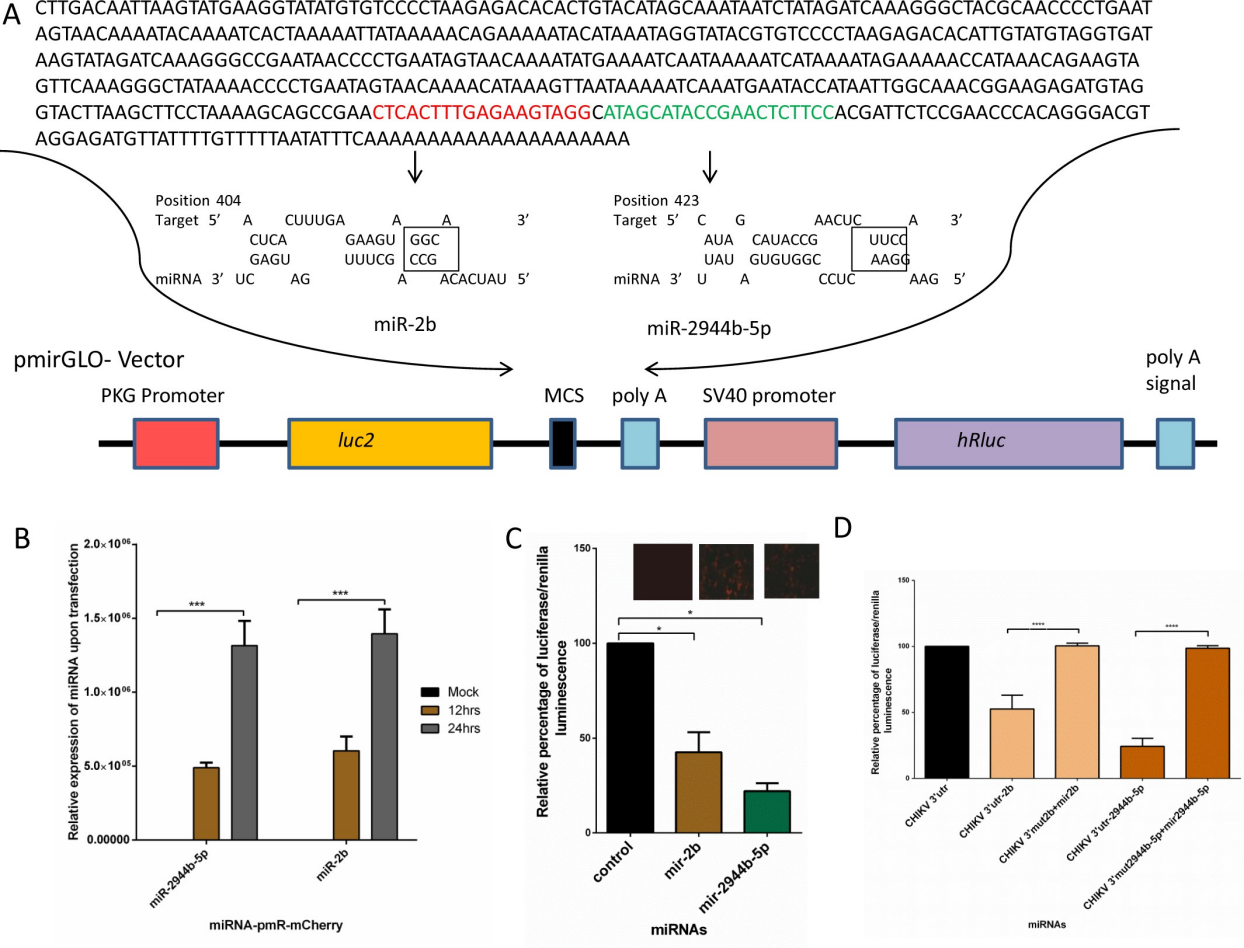
Luciferase assay for miRNAs to CHIKV 3′ UTR binding. (A) Sequence of CHIKV 3' UTR cloned into the pmirGLO vector, and miRNA-3'UTR binding with mutations on boxed sequence of 3'UTR, where sequence was replaced with nucleotide Adenine. (B) Relative expression of miRNAs miR-2944b-5p and miR-2b cloned into pmR-mCherry, at 12hrs and 24hrs post transfection into HEK-293T. (C) Luciferase assay for miR-2b and miR-2944b-5p binding to 3' UTR. (D) Luciferase assay showing binding of miR-2b and miR-2944b-5p to 3' UTR with their mutated (boxed sequence of seed region) binding site. Empty pmirGLO vector along with miR-2944b-5p cloned in pmR-mCherry vector was taken as control. The experiments were repeated at least thrice and each experiment included at least three treatments Data are expressed as mean ± SEM, *P < 0.05, ****P < 0.0001.

Several studies have established that viruses contain binding sites for host miRNAs in their UTRs [10,27,28]. In order to explore whether *Aedes* miRNAs do have binding sites in the CHIKV 3′ UTR region, we identified two miRNAs, namely, miR-2944b-5p and miR-2b, that were most significantly regulated upon CHIKV infection in a previous study [23], and set out to perform the Dual-Glo luciferase assay using the pmR-mCherry and pmirGLO system (Fig 1A). To confirm the expression of miRNAs after transfection we performed the qRT-PCR (Fig 1B). The assays revealed that miR-2b and miR-2944b-5p exhibited a significant level of binding to CHIKV 3′ UTR, with 60% and 80% reduction in relative percentage of Luciferase/Renilla luminescence, respectively (Fig 1C). Also, to confirm if binding to CHIKV 3'UTR is specific to miR-2944b-5p we also selected another miRNA, namely, miR-13-3p, having no predicted binding site at CHIKV 3'UTR, as a negative control, to perform luciferase assay, and results showed that mir-2944b-5p is specific in binding to its target region in CHIKV-3'UTR (**S1 Fig**). The assay further showed that the relative percentage of luciferase/renilla luminescence of miR-2944b-5p was slightly higher than that of miR-2b. Further, to confirm that these are the binding sites of seed regions of miRNAs, we mutated the binding sites of CHIKV 3′ UTR and performed luciferase assays. Reversal of luciferase expression in the mutated clones confirmed the role of these sites in the UTR–miRNA interaction (Fig 1D).

### miR-2944b-5p significantly regulates the viral infection in the Aag2 cell line as compared to miR-2b, and controls CHIKV replication in *Ae*. *aegypti*

**Fig 2:**
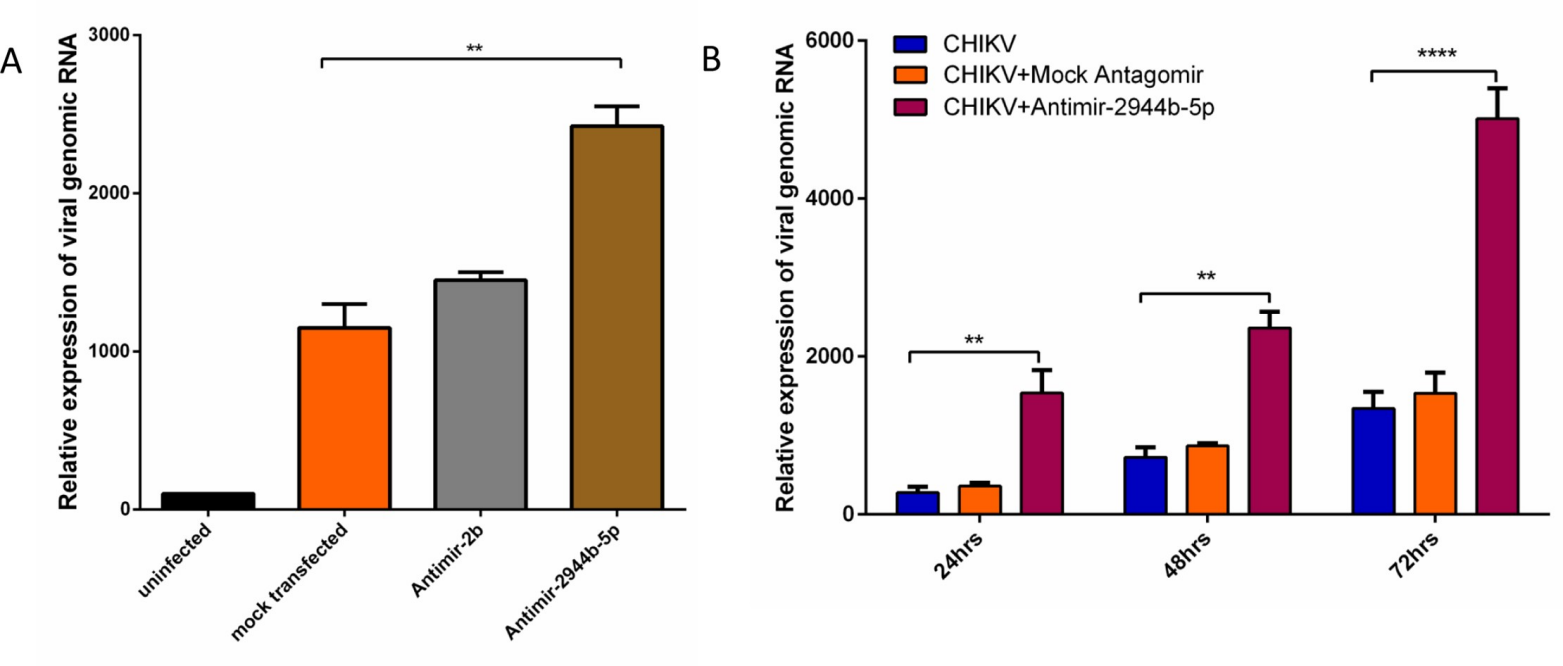
Effect of miRNA on viral replication using qRT-PCR. (A) Expression level of CHIKV genomic RNA after 24 hrs post infection (MOI 1) and 48 hrs post antagomir transfection for miR-2944b-5p and miR-2b, relative to uninfected cells. The mock transfected contained scrambled miRNA. (B) QRT-PCR analysis of CHIKV genomic RNA in *Ae*. *aegypti* mosquitoes under different conditions (CHIKV infection, CHIKV infection with scrambled miRNA (mock), and CHIKV infection with Antimir-2944b-5P) and at different time points, 24hrs, 48hrs, and 72hrs post infection. The experiments were repeated at least thrice and each experiment included at least three treatments Data are expressed as mean ± SEM,**P < 0.01 ***P < 0.001.

Further to test whether the binding of miRNAs to CHIKV 3′ UTR had any functional relevance to the CHIKV infection, we silenced these miRNAs using antagomir (miRNA inhibitor) in *Ae*. *aegypti* cells and infected them with CHIKV to check for any changes in virus replication. Optimum silencing of the miRNAs was achieved at 24 hrs post-transfection (**S2 Fig**), and when the cells were infected with CHIKV (MOI 1) 24 hrs post-silencing, we observed that at 24 hrs post infection CHIKV replication increased more than two fold, (*P*<0.05), when miR-2944b-5p was silenced (Fig 2A). These results were promising enough to hypothesize that miR-2944b-5p was probably playing a more direct role in CHIKV replication in *Ae*. *aegypti*. After confirming the regulation of CHIKV replication by miR-2944b-5p in Aag2 cells, *Ae*. *aegypti* mosquitoes were used for further validation. The *Ae*. *aegypti* mosquitoes were injected with Anitimir-2944b-5p to silence miR-2944b-5p, also scrambled miRNA served as a mock. Post miR-2944b-5p silencing in four-day-old mosquitoes, we infected the mosquitoes by feeding them with CHIKV-spiked defibrinated blood. After CHIKV infection, the mosquitoes were collected at different time points, 24hrs, 48hrs, 72hrs for RNA isolation and qRT-PCR analysis. Monitoring CHIKV replication in mosquitoes in which miR-2944b-5p was knocked down revealed, a three fold, increase (*P*<0.0001) in CHIKV infection than that observed in the cell lines (Fig 2B), thereby confirming that the miRNA was playing an important role in CHIKV replication in *Ae*. *aegypti*.

### *Ae*. *aegypti* transcript vps-13 is a target of miR-2944b-5p

**Fig 3:**
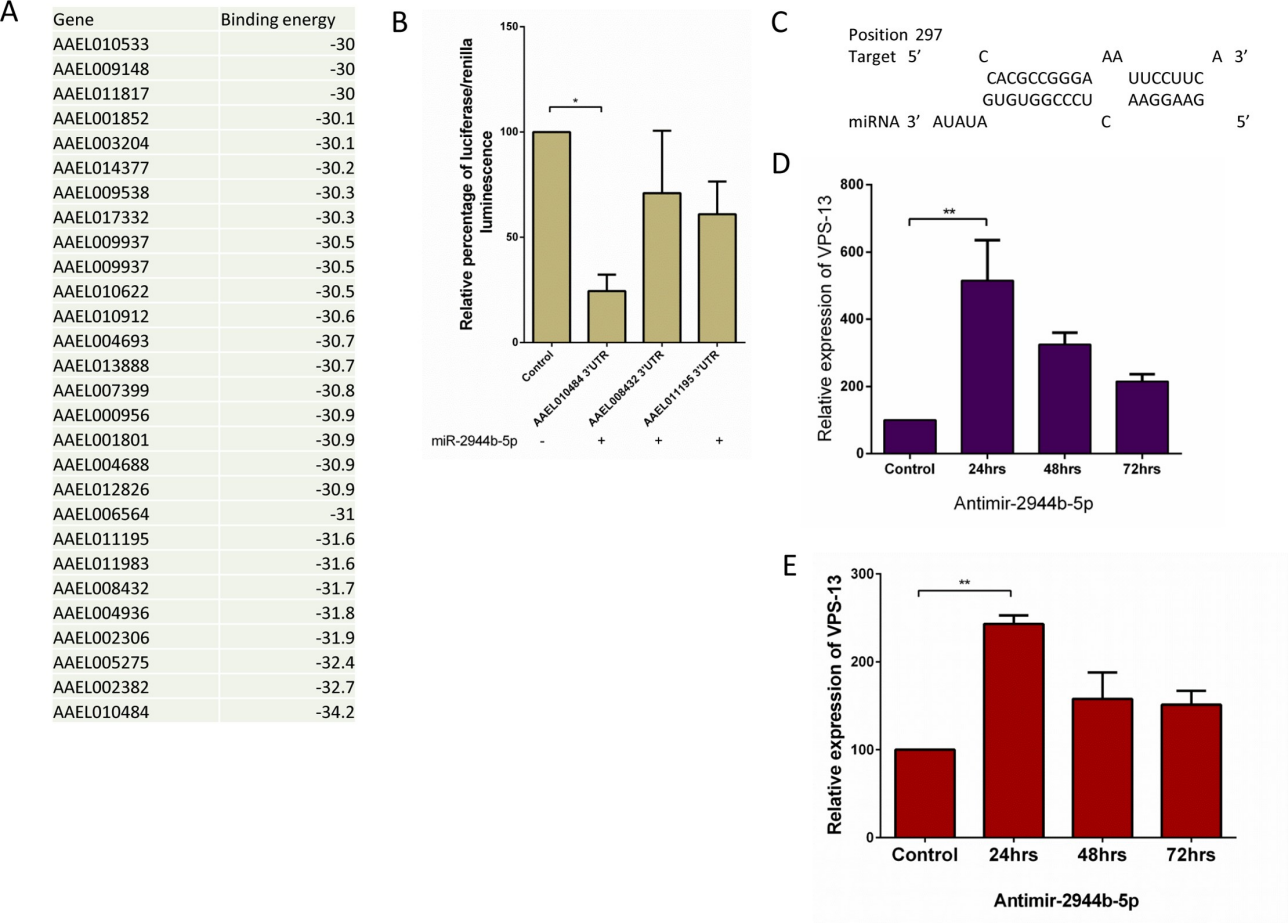
*Ae*. *aegypti* cellular target prediction and validation. (A) List of top 28 predicted cellular targets for miR-2944b-5p using RNAhybrid tool. (B) Luciferase assay shows relative percentage of luciferase/renilla luminescence for miR-2944b-5p binding to its three targets (AAEL011195, AAEL008432, and AAEL010484), Empty pmirGLO vector along with miR-2944b-5p cloned in pmR-mCherry vector was taken as control (C) Binding region between vps-13 and miR-2944b-5p. (D) Relative expression of vps-13 at different time points 24hrs, 48hrs, 72hrs during miR-2944b-5p inhibition in Aag2 cell line using Antimir (E) Relative expression of vps-13 at different time points 24hrs, 48hrs, 72hrs during miR-2944b-5p inhibition in *Ae*. *aegypti* using Antimir. The experiments were repeated at least thrice and each experiment included at least three treatments. Data are expressed as mean ± SEM, *P < 0.05.

Target prediction of miR-2944b-5p amongst *Ae*. *aegypti* transcripts revealed a total of 28 putative targets based on the selection criteria (Fig 3A). Three most significant putative targets were taken for validation based on their statistical significance (p≤0.001) and free energy cutoff -31kcal/mol. We performed luciferase assay to confirm the binding of miR-2944b-5p towards these three annotated targets. The result showed AAEL010484 (vps-13) with more than 75% reduction in the relative percentage of luciferase/renilla luminescence and was taken forward for further validations (Fig 3B and 3C). Furthermore, the expression level of vps-13 was observed to be affected with the miR-2944b-5p inhibition using Antimir-2944b-5p in Aag2 cell line and *Ae*. *aegypti* mosquito(Fig 3D and 3E). The expression level of miR-2944b-5p was also analyzed at different time point after the inhibition using Antimir **(S3 Fig).**

### VPS-13 controls CHIKV replication in Aag2 cell line and *Ae*. *aegypti*

**Fig 4:**
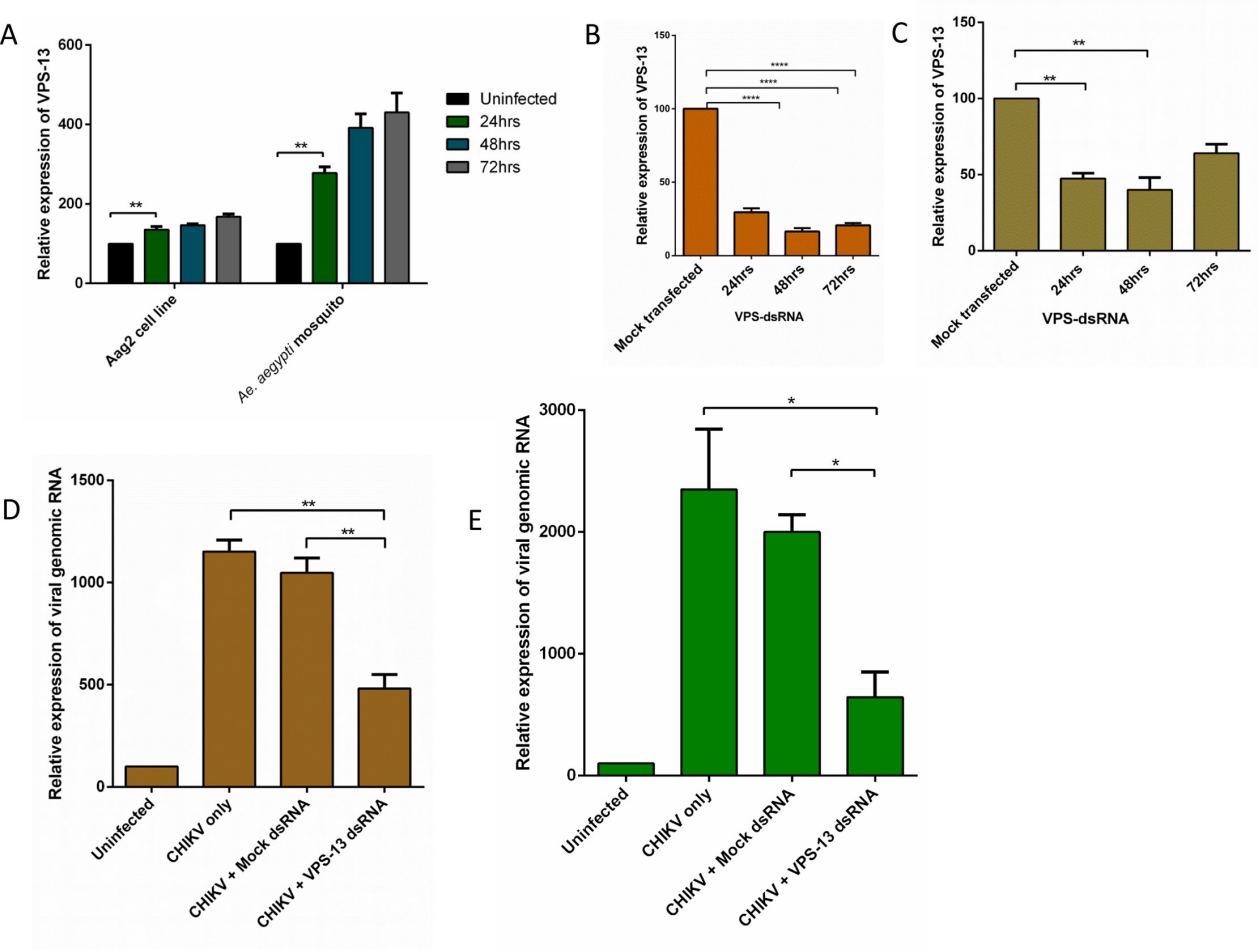
vps-13 and CHIKV infection in Aag2 cells. (A) Relative expression vps-13 during CHIKV infection at different time points, 24hrs, 48hrs and 72hrs in Aag2 cell line and *Ae*. *aegypti* mosquito. (B) Relative expression of vps-13 upon dsRNA transfection at different time points 24hrs, 48hrs and 72hrs in Aag2 cell line and (C) *Ae*. *aegypti* mosquito, mock contained the gfp dsRNA as negative control. (D) Relative expression of viral genomic RNA in Aag2 cell line and (E) *Ae*. *aegypti* mosquito upon vps-13 inhibition through dsRNA at 24hrs post infection (cells were infected after 24hrs of dsRNA transfection and mock dsRNA contained gfp dsRNA as a negative control). The experiments were repeated at least thrice and each experiment included at least three treatments. Data are expressed as mean ± SEM, ****P < 0.0001, **P < 0.01, *P < 0.05.

Relative expression of vps-13, at different time points post CHIKV infection, were checked in Aag2 cell line and *Ae*. *aegypti* mosquito. The results showed more than two fold increase in the level of vps-13 suggesting the role of vps-13 in CHIKV replication (Fig 4A). Relative expression of vps-13 in Aag2 cell line and *Ae*. *aegypti* mosquito at different time points upon vps-13 dsRNA transfection showed the inhibition and fall in the expression level of vps-13 which was used to study the CHIKV infection level in next experiment (Fig 4B and 4C). Relative expression of CHIKV genomic RNA decreased 24hrs post infection when vps-13 is silenced by dsRNA in Aag2 cells and *Ae*. *aegypti* mosquito. For negative control control gfp dsRNA was used (Fig 4D and 4E). Collectively all this data showed the regulation of vps-13 on CHIKV replication.

### vps-13 interacts with miR-2944b-5p to modulate CHIKV replication

**Fig 5:**
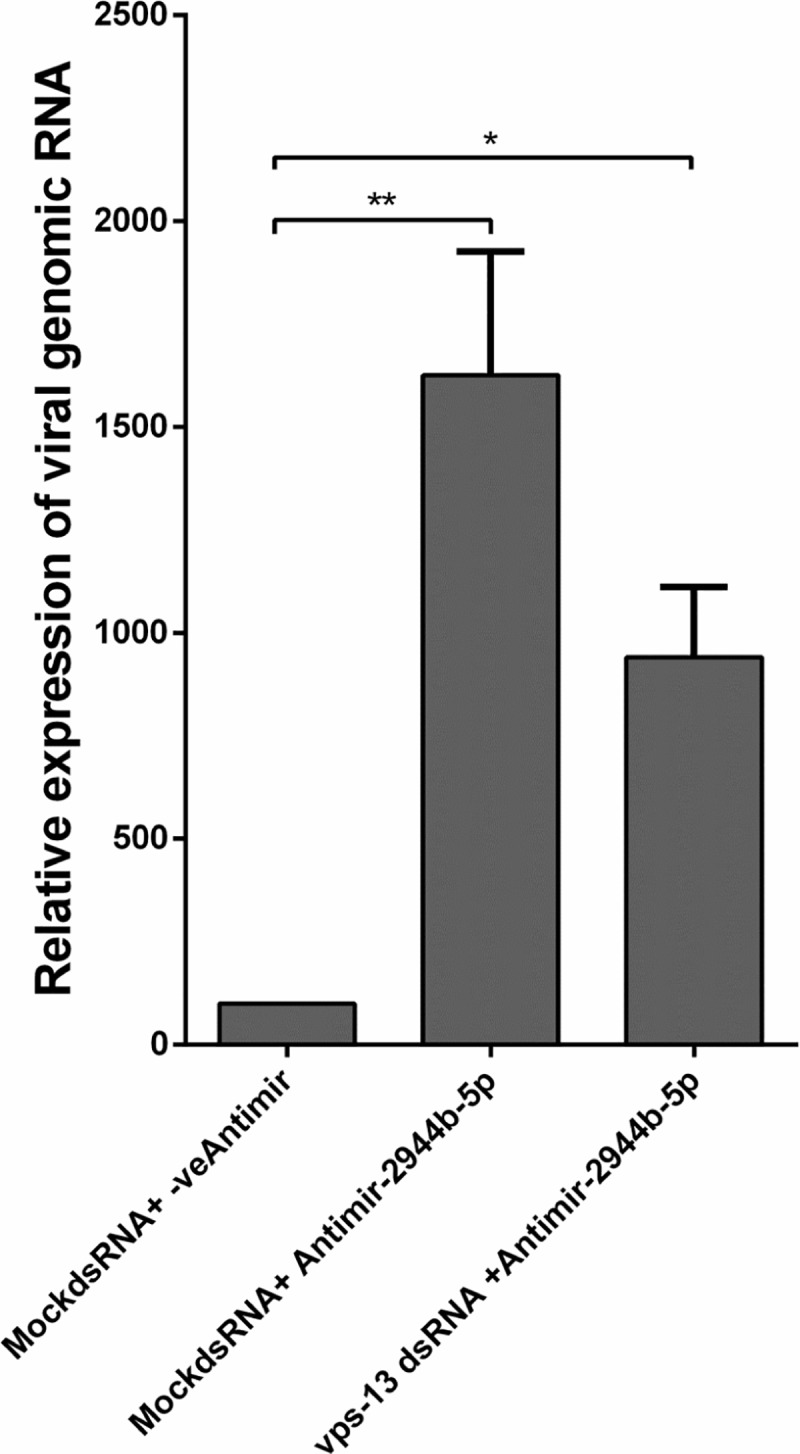
Effect of simultaneous inhibition of miR-2944b-5p and vps-13 during CHIKV infection in Aag2 cells. The experiments were repeated at least thrice and each experiment included at least three treatments. Data are expressed as mean ± SEM, **P < 0.01, *P < 0.05.

Finally, we wanted to evauate the independent roles of miR-2944b-5p and its target, vps-13 on CHIKV replication in *Ae*. *aegypti* cells. For this purpose, we silenced vps-13 and miR-2944b-5p simultaneously and studied the impact of silencing on CHIKV replication in Aag2 cells. As described in the previous sections, 2944b-5p inhibition increased the CHIKV replication significantly. Simultaneous silencing of miR-2944b and vps-13 however revealed that CHIKV replication decreased as compared to when only miR-2944b-5p was silenced, thereby suggesting the vps-13 could be modulating CHIKV replication by interacting with miR-2944b-5p (Fig 5). This modulation of CHIKV replication by vps-13 and miR-2944b-5p interplay possibly helps persistent CHIKV infection in *Ae*. *aegypti* and allowing the cells to survive longer.

### CHIKV infection affects MMP in *Ae*. *aegypti* cells

**Fig 6:**
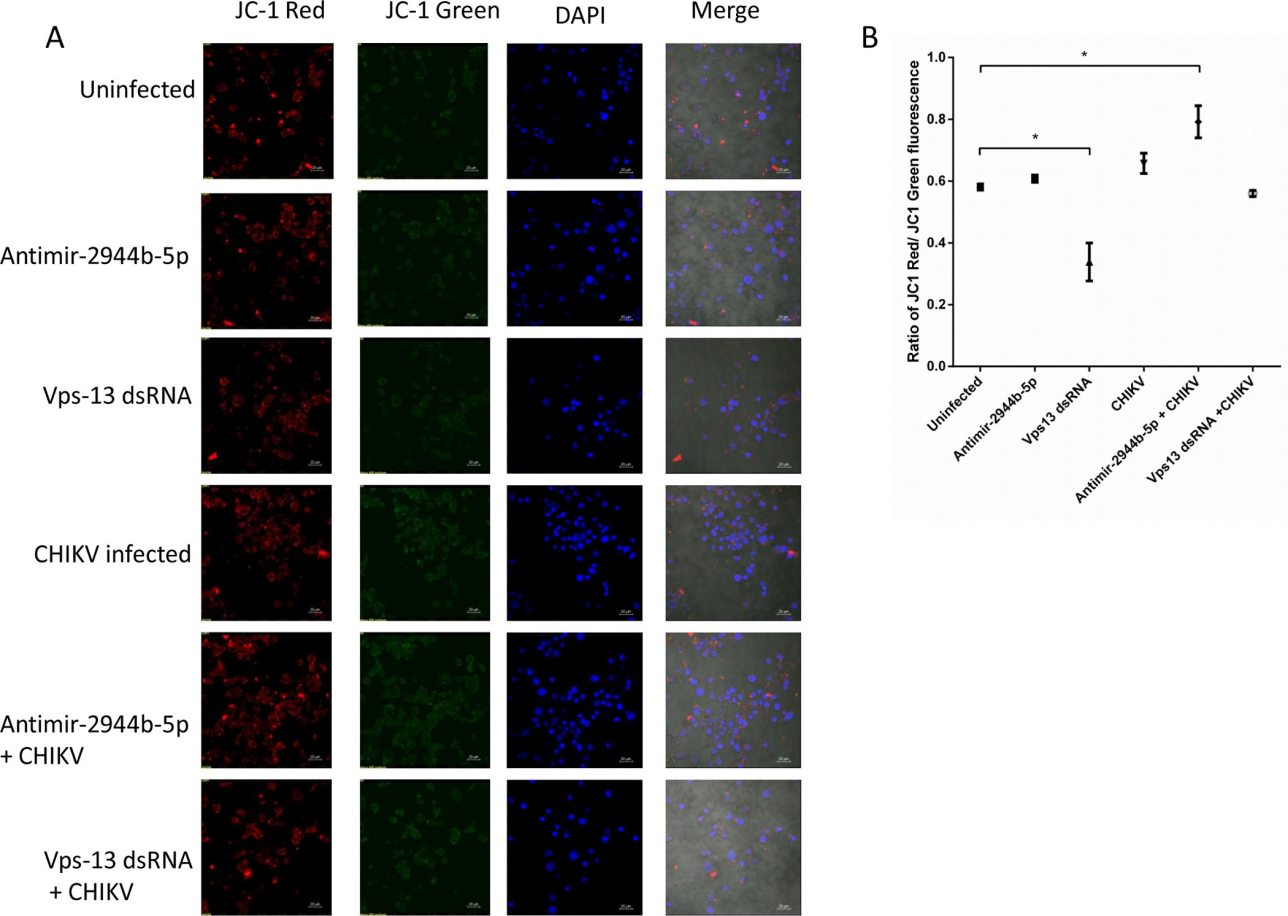
Effect of CHIKV, Antimir-2944b-5p and vps-13 on MMP in the Aag2. (A) The MMP in the Aag2 cell line. After 24 hrs post transfections (with Antimir-2944b-5p and vps-13 dsRNA) cells were infected with CHIKV (MOI 1). After 24 hrs post infection, the cells were stained with the mitochondrial-selective JC-1 dye for mitochondrial potential and DAPI to stain nuclei for confocal imaging analysis. (B) The ratio of JC-1 red and JC-1 green fluorescence was measured and represented as histogram. The experiments were repeated at least twice and each experiment included at least three treatments. ***P < 0.001, *P < 0.05.

After confirming the role of miR-2944b-5p and its target vps-13 in controlling CHIKV replication, we were intrigued to understand the underlying mechanism by which this miRNA and its target could regulate CHIKV replication in *Ae*. *aegypti*. Analysis of all the cellular targets of the miR-2944b-5p, including vps-13, revealed that most of the targets were prominently involved in regulating mitochondrial functions. It is known that CHIKV induces severe oxidative stress in the host it infects. Among the several organelles involved in regulating the cellular processes during viral replication and in combating oxidative stress, mitochondria are known to play a critical role [29,30]. Considering its importance in CHIKV infection, we decided to evaluate MMP in the Aag2 cells. The MMP was evaluated by the ratio of the mitochondrial dye, JC-1 based on its ability to enter the mitochondria that is depicted as red fluorescence vs its availability in the cytoplasm as seen as green fluorescence. A higher ratio of red JC-1 by green JC-1 depicts an increase in MMP, which in turn reveals better mitochondrial integrity. The confocal microscopy results clearly showed that vps-13 had a significant role in maintenance of MMP in *Ae*. *aegypti* cells. Also, CHIKV infection did not hamper the cellular MMP clearly emphasizing that CHIKV is not pathogenic in *Ae*. *aegypti* cells. Upon miR-2944b-5p silencing in uninfected cells, we did not observe a significant increase in cellular MMP. However, miR-2944b-5p silencing and subsequent CHIKV infection revealed an increase in the cellular MMP, leading us to believe that the miRNA has a direct role to play during CHIKV infection. Further, our data (Fig 4A) revealed that there was an overexpression of vps-13 upon CHIKV infection. Silencing of vps-13 and subsequent CHIKV infection, revealed an increase in the cellular MMP albeit marginally (Fig 6A and 6B).

## Discussion

The present study provides evidence for the functional relevance of a mosquito miRNA binding to the 3′ UTR of an arbovirus and regulating its replication in the insect cells. It is known that miRNAs are regulatory in their function and participate in several cellular processes such as development, reproduction, and immunity [31–34]. Recent studies have shown that apart from these cellular functions, host miRNAs control translation and replication of viruses through binding to the 5' UTR [15,20,35], 3' UTR as well as coding regions of viral genome [36]. All these studies have reported miRNAs interacting with human pathogenic viruses of both DNA and RNA origin. However, studies on miRNA interactions with arboviruses that utilize different hosts for their propagation and transmission are scant [23,24]. Owing to the distinct defense mechanisms between the mammalian and insect systems, it is of immense interest to understand how arboviruses are able to survive in such diverse environments, which was the premise of our present study.

RNA viruses have a high mutation rate due to the absence of proofreading ability [37]. In spite of this, these viruses do retain miRNA binding sites within their genome under selective pressure, where downregulating its own replication is necessary for persistent infection [38,39]. As seen in the present study, *Ae*. *aegypti* miRNAs were found to possess binding sites in the 3′ UTR of CHIKV and examination of the functional relevance of this binding proved that miRNAs interfere with viral replication using completely different mechanisms. A recent study from our laboratory showed that the *Ae*. *aegypti* miRNA, miR-2b regulated CHIKV replication by regulating its cellular target, ubiquitin related modifier [40]. The present study additionally revealed that miR-2b bound to CHIKV 3'UTR, albeit not having much biological relevance in directly controlling CHIKV replication. However, the present study has thrown light on another *Aedes* miRNA, miR-2944b-5p, in controlling CHIKV replication in a more direct manner. Furthermore, this study shows that regulation of viral replication by *Aedes* miRNA is not a straightforward phenomenon; rather a complex interaction involving the mosquito’s miRNA, its targets and the virus that results in modulating the cell’s function, in this case, favorably for the virus. Our results are in concurrence with similar findings in other studies involving arboviruses, where in a study miR-142-3p restricts the replication of the North American (NA) eastern equine encephalitis virus (EEEV) [35]. These recent in-depth analyses of *Aedes* miRNAs reveal that there is a strong level of interaction between the mosquito miRNAs and arboviruses either directly, as seen in the present study, or through their targets as recorded elsewhere [41].

In addition to viruses possessing binding sites to vector miRNAs, another important aspect that deserves rationalization is the length and structure of the viral 3′ UTR. Among the genotypes of CHIKV, it is known that the length of the 3′ UTR varies and has a direct implication on CHIKV inter-genotypic virulence in the vector [18]. Analysis of miR-2944b and miR-2b binding with 3′ UTRs of all the CHIKV lineages revealed differences in the binding energies (**S4 Fig**). In the Asian lineage of CHIKV, duplication has been shown to occur in the 3’UTR [18], and our analysis reveal that miR-2b and miR-2944b-5p possessed two binding sites at the point of duplication thereby reducing the level of confidence of its binding capacity in other lineage of CHIKV. The present study was performed using the Indian Ocean island lineage and still had significant effect of CHIKV replication. These observations prompt us to believe that these virus–vector interactions are specific and pivotal for the extent of the pathogenicity of CHIKV and warrants further in-depth studies utilizing strains from all CHIKV lineages.

Analysis of miR-2944b-5p across the kingdom revealed that this miRNA was restricted to the insects and that it was not present in the mammals. This prompted us to hypothesize that this miRNA could be involved in a vector-specific function with respect to CHIKV replication and might play a role to differentiate some survival feature specifically imparted to mosquito vectors. Analysis of its targets revealed that this miRNA was involved in regulation to mitochondrial functions and membranes. Amongst the significant targets, presence of vps-13 led us to hypothesize the possible involvement of mitochondria during CHIKV infection. In previous studies vps-13 has been shown to have role in maintaining the function of mitochondria and membrane morphogenesis in other systems [42,43]. It has been prominently shown that mitochondria are organelles which play a critical role during virus infection [29,44]. They are vital for energy production and oxidative phosphorylation and are also reported to participate in an array of cellular functions including production of reactive oxygen species [45], apoptosis [46], and calcium homeostasis [47]. Many of the above-mentioned phenomena are affected upon virus infection in a cell. It has been reported that during the early stages of viral replication, some viruses try to avoid apoptosis so as to efficiently replicate [48]. Analysis of MMP during CHIKV replication in vector cells in the present study revealed that the MMP was affected as virus replication progressed. In the case of mosquito cells, mitochondrial integrity is regulated and maintained in the presence of CHIKV and vps-13 plays role in controlling it. These findings suggest that either the mosquito cells have defense mechanisms that regulate viral growth or the virus could be manipulating the mosquito cells to ensure their survival within the cells, in any case vps-13 plays a significant role in manipulating the virus replication.

**Fig 7:**
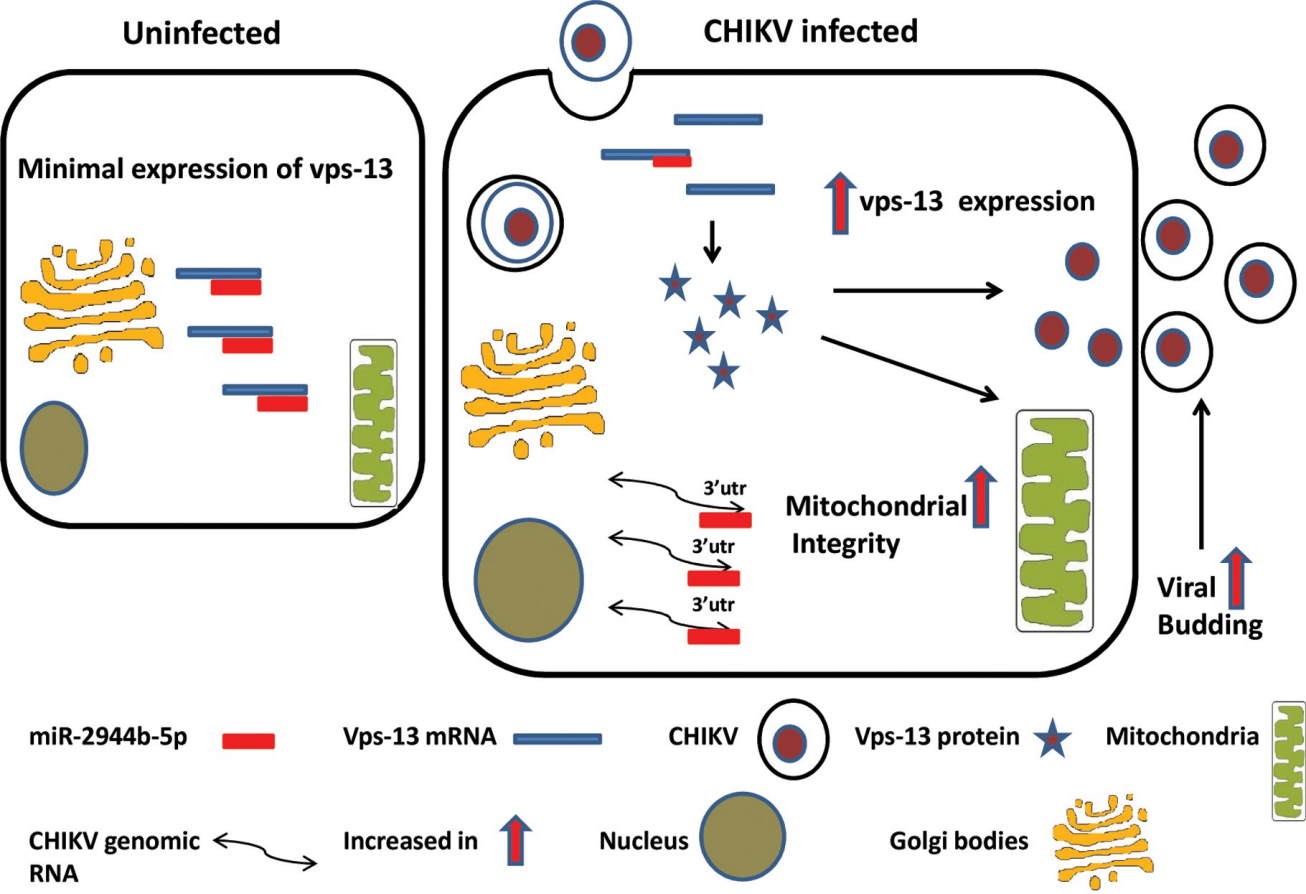
Proposed model for the miR-2944b-5p interplay between CHIKV genome and mitochondrial integrity.

Several reports have highlighted the role of miRNAs in regulating the function, metabolism, and morphology of mitochondria [49–51]. In the present study, miR-2944b-5p seems to be involved in stabilizing the cells during CHIKV infection in Aag2 cells. As evidenced by the miRNA profiling during CHIKV infection in *Ae*. *aegypti* cells [23], miR-2944b-5p gets over-expressed during CHIKV replication **(S5 Fig)** and this over-expression could be related to its role in stabilizing *Ae*. *aegypti* cells during CHIKV infection. Based on our findings, we propose a mechanism explaining the role of miR-2944b-5p in maintaining CHIKV in mosquito cells wherein, upon CHIKV infection, miR-2944b-5p is differentially regulated and helps in maintaining the integrity of mitochondria involving vps-13 to help persistent virus replication (Fig 7).

The present study provides evidence for direct binding of an *Aedes* miRNA, miR-2944b-5p to the 3′ UTR of CHIKV. The study also stipulates a possible role of miR-2944b-5p and vps-13 in maintaining the MMP in a cell infected with CHIKV. It is quite possible that there may be other cellular factors that may be involved in the execution of this feature; nevertheless, our results provide strong evidence for the role of *Aedes* specific miRNAs and cellular targets in the phenomenon of CHIKV infection in *Aedes* cells. One novel strategy for translating these findings to address public health concerns is to take advantage of the fact that this miRNA is insect specific and is not present in the mammalian systems, thereby making it a good candidate for regulating CHIKV infection in the humans; however, further in-depth studies are essential to address this hypothesis.

## Supporting information

S1 Table Primer details. Sequences of the primers used for qRT-PCR analysis.
(PDF)

S1 Fig Relative percentage of luciferase/renilla luminescence of miR-2944b-5p and miR-13-3p. (TIF)

S2 Fig Relative expression of miRNA upon transfection of Antimir-2944b-5p and Antimir-2b in Aag2 cell line. (TIF)

S3 Fig Relative expression of miRNA upon transfection of Antimir-2944b-5p in *Ae. aegypti* mosquito. (TIF)

S4 Fig Binding site for miR-2944b-5p and miR-2b in 3’ UTR of Asian lineage and ECSA lineage (CHIKV: Line diagram). The sites with variations are shown with a “star”. The yellow box highlights the duplication site whereas the blue box is the retained sequence of 3'UTR. The minimum free binding energies (mfe) are also shown along with the binding sites.
(TIF)

S5 Fig Fold change of miRNA-2944b-5p at different time points upon CHIKV infection. (TIF)
