# Supplementary material for: Aedes aegypti microRNA, miR-2944b-5p interacts with 3'UTR of chikungunya virus and cellular target vps-13 to regulate viral replication
Source: PLoS Negl Trop Dis. 2019 Jun 5;13(6):e0007429. doi: 10.1371/journal.pntd.0007429 (PMC6576790; doi:10.1371/journal.pntd.0007429)
Supplement: S1 Table — Sequences of the primers used for qRT-PCR analysis. (PDF) [file pntd.0007429.s001.pdf]

Supplementary Table 1. Details of primers. Table shows the sequences of the qRT-PCR primers synthesised

Name	Fwd sequence	Rev sequence
miR-2b	TATCACAGCCAGCTTTGATG	Universal
miR-2944b-5p	GAAGGAACUCCCGGUGUGAUAUA	Universal
CHIKV E1	TACCCATTTATGTGGGGC	GCCTTTGTACACCACGATT
5.8s	CCCTAGGCAGGGGATCACT	GGCACTCAAGAATGTGTGCAT
Vps-13	ATTCGCAGGTGTCGCTATGG	GGTTCCGAGGCAATCTTCGT
