# Supplementary figures and images for: Aedes aegypti microRNA, miR-2944b-5p interacts with 3'UTR of chikungunya virus and cellular target vps-13 to regulate viral replication
Source: PLoS Negl Trop Dis. 2019 Jun 5;13(6):e0007429. doi: 10.1371/journal.pntd.0007429 (PMC6576790; doi:10.1371/journal.pntd.0007429)

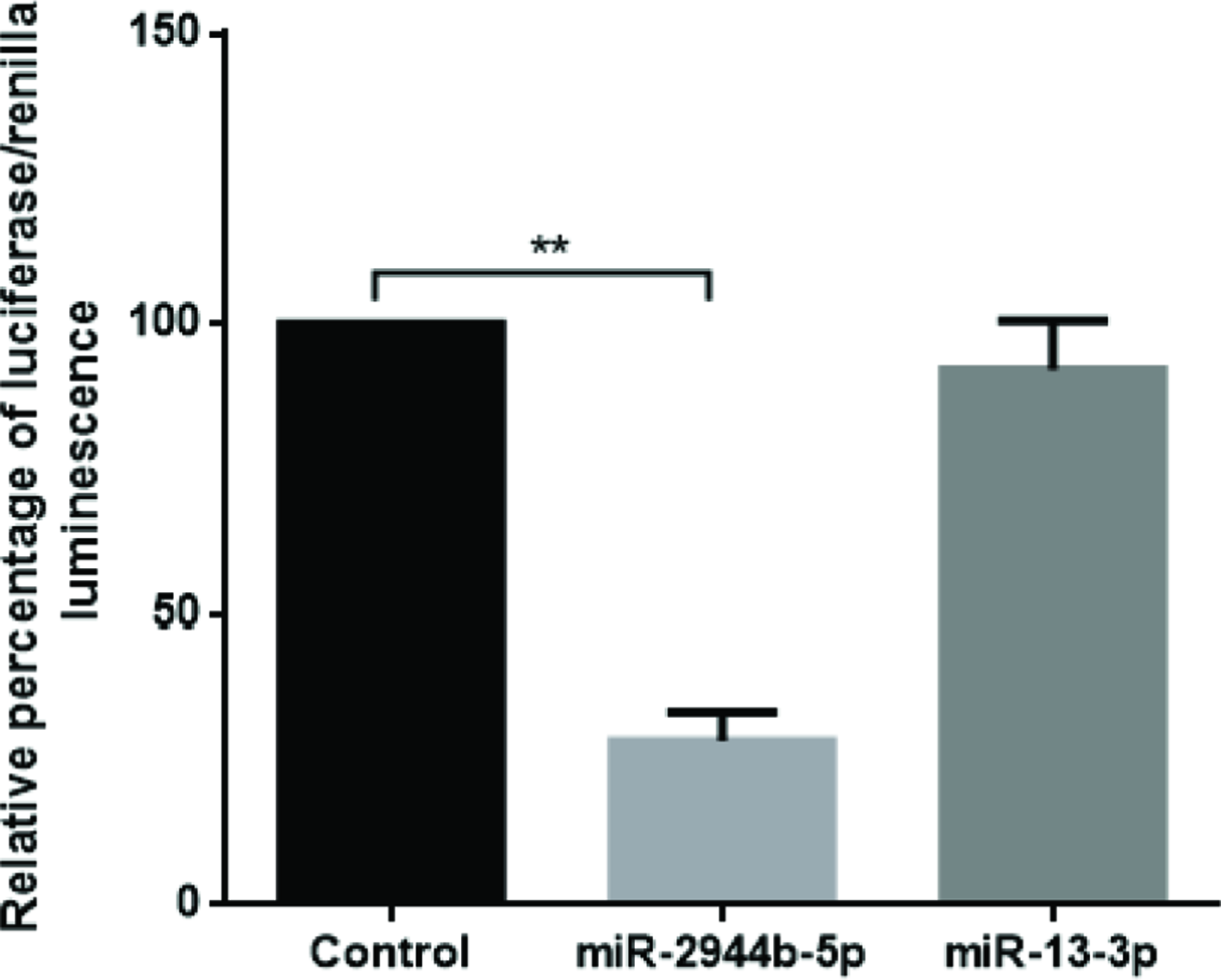

Supplement: S1 Fig — (TIF) [file pntd.0007429.s002.tif]

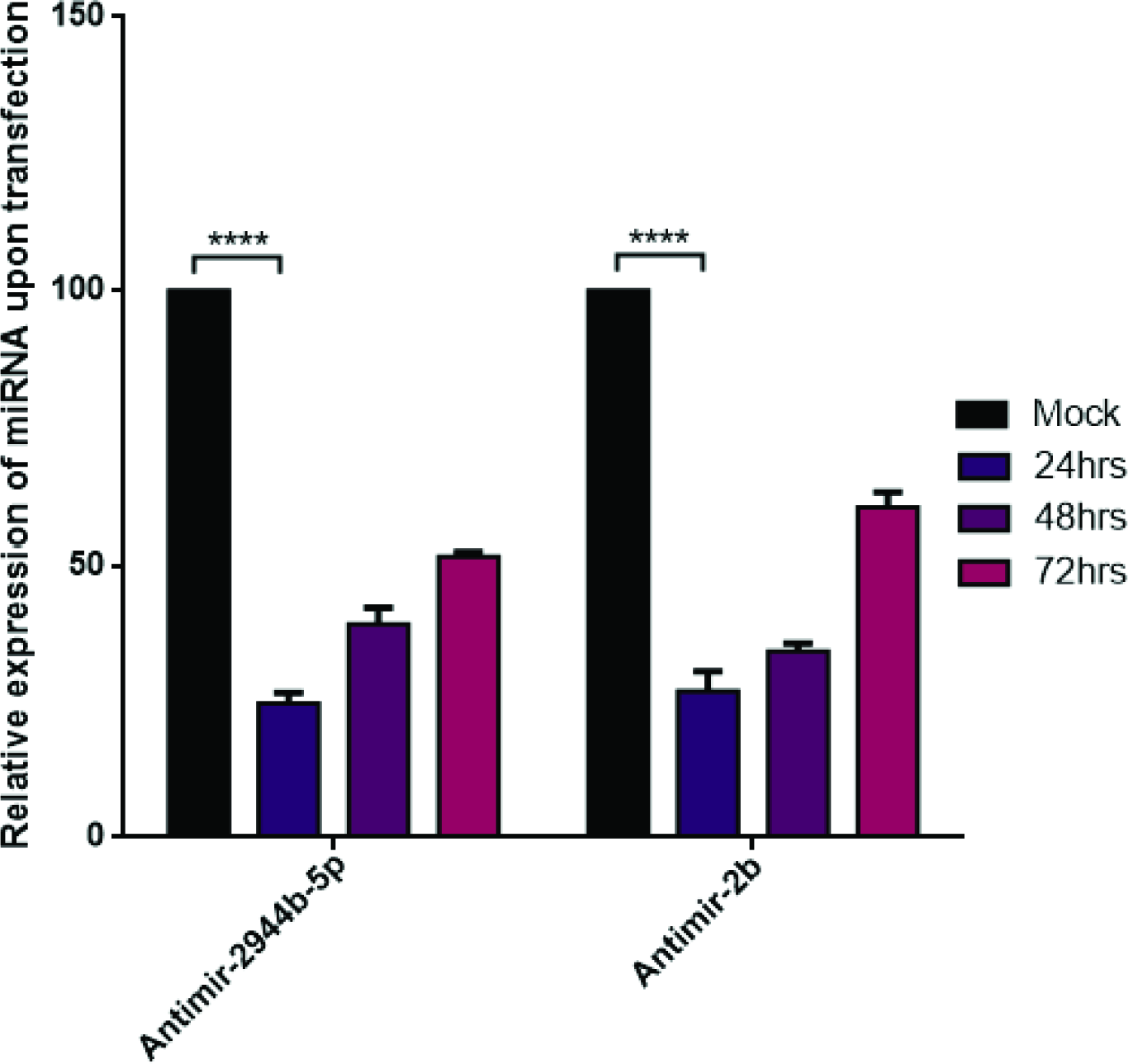

Supplement: S2 Fig — (TIF) [file pntd.0007429.s003.tif]

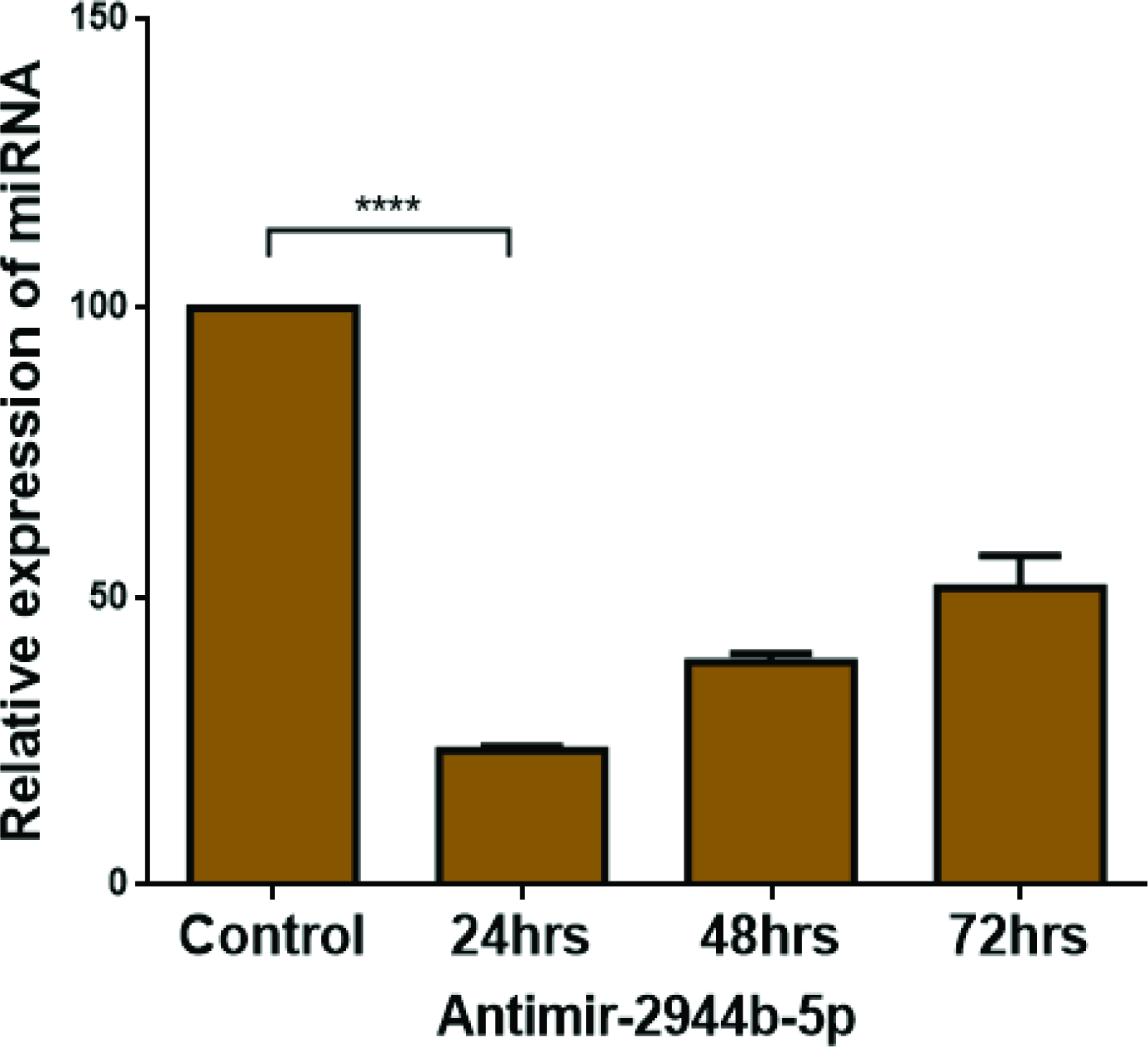

Supplement: S3 Fig — (TIF) [file pntd.0007429.s004.tif]

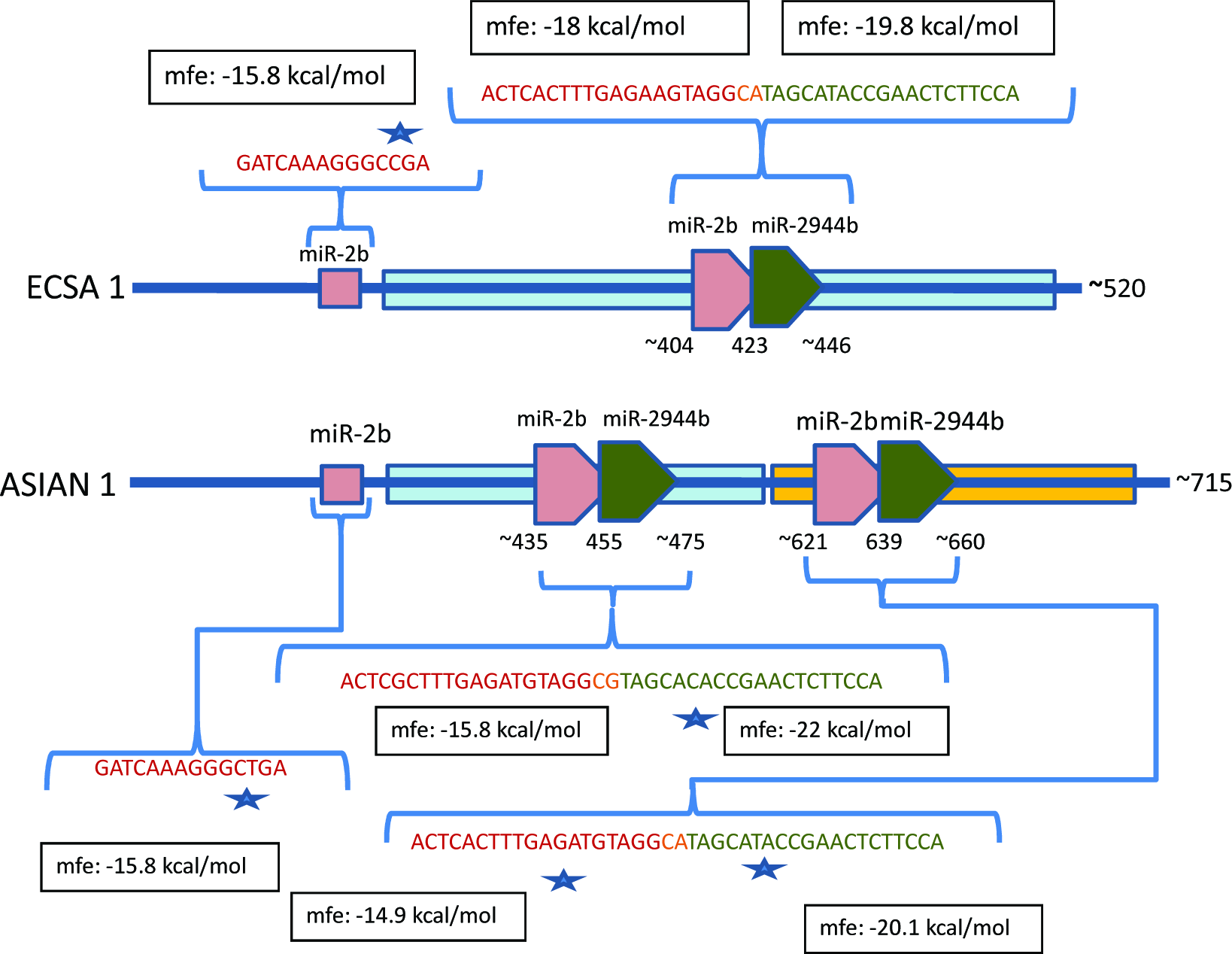

Supplement: S4 Fig — The sites with variations are shown with a “star”. The yellow box highlights the duplication site whereas the blue box is the retained sequence of 3'UTR. The minimum free binding energies (mfe) are also shown along with the binding sites. (TIF) [file pntd.0007429.s005.tif]

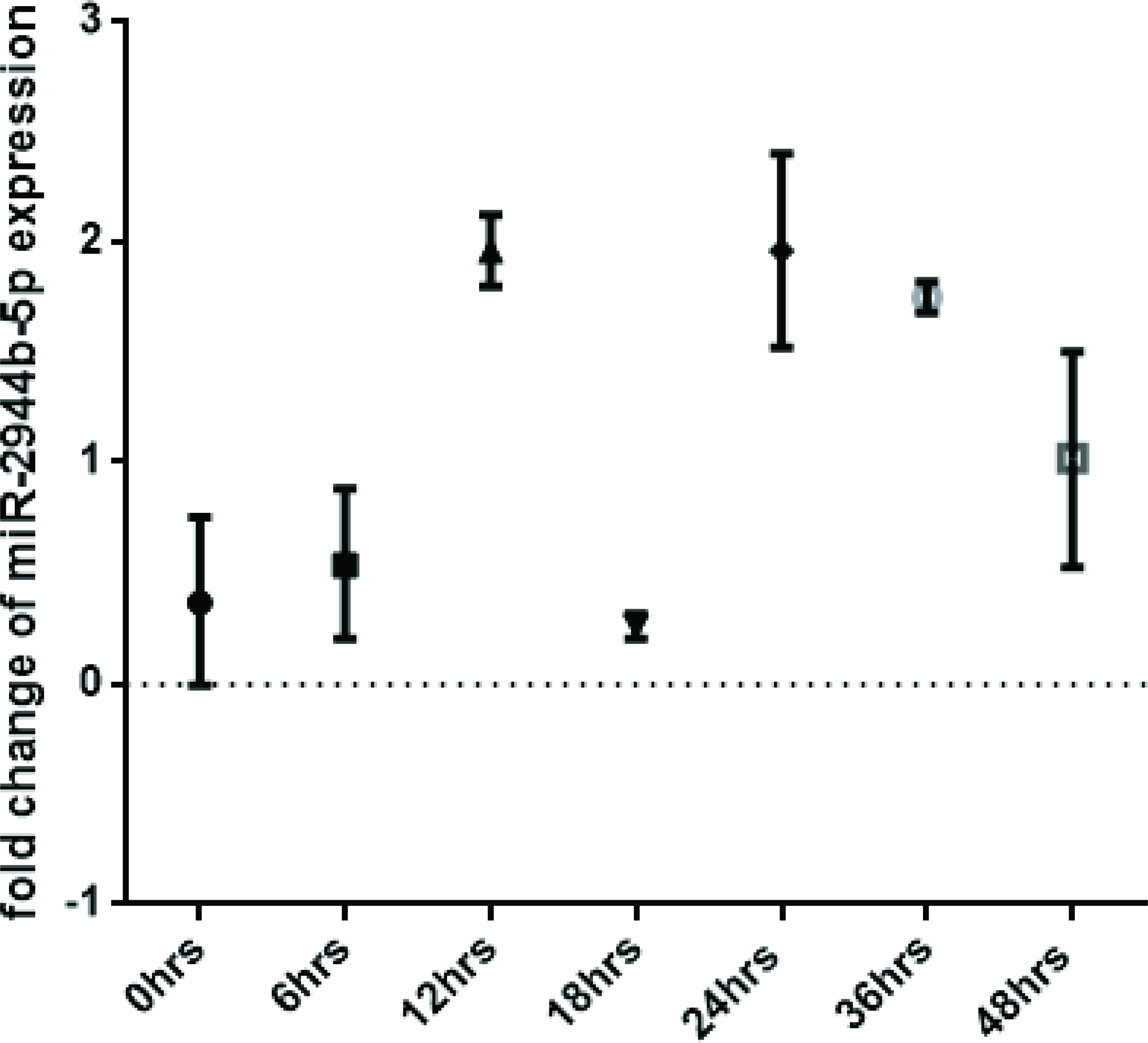

Supplement: S5 Fig — (TIF) [file pntd.0007429.s006.tif]
